# Nanoparticle-Delivered Multimeric Soluble CD40L DNA Combined with Toll-Like Receptor Agonists as a Treatment for Melanoma

**DOI:** 10.1371/journal.pone.0007334

**Published:** 2009-10-08

**Authors:** Geoffrey W. Stone, Suzanne Barzee, Victoria Snarsky, Camila Santucci, Brian Tran, Robert Langer, Gregory T. Zugates, Daniel G. Anderson, Richard S. Kornbluth

**Affiliations:** 1 Department of Medicine, University of California San Diego, La Jolla, California, United States of America; 2 Department of Medicine, VA San Diego Healthcare System, La Jolla, California, United States of America; 3 David H. Koch Institute for Integrative Cancer Research at MIT, Cambridge, Massachusetts, United States of America; 4 Chemical Engineering Department, Massachusetts Institute of Technology, Cambridge, Massachusetts, United States of America; University of Nebraska, United States of America

## Abstract

Stimulation of CD40 or Toll-Like Receptors (TLR) has potential for tumor immunotherapy. Combinations of CD40 and TLR stimulation can be synergistic, resulting in even stronger dendritic cell (DC) and CD8+ T cell responses. To evaluate such combinations, established B16F10 melanoma tumors were injected every other day X 5 with plasmid DNA encoding a multimeric, soluble form of CD40L (pSP-D-CD40L) either alone or combined with an agonist for TLR1/2 (Pam_3_CSK_4_ ), TLR2/6 (FSL-1 and MALP2), TLR3 (polyinosinic-polycytidylic acid, poly(I:C)), TLR4 ( monophosphoryl lipid A, MPL), TLR7 (imiquimod), or TLR9 (Class B CpG phosphorothioate oligodeoxynucleotide, CpG). When used by itself, pSP-D-CD40L slowed tumor growth and prolonged survival, but did not lead to cure. Of the TLR agonists, CpG and poly(I:C) also slowed tumor growth, and the combination of these two TLR agonists was more effective than either agent alone. The triple combination of intratumoral pSP-D-CD40L + CpG + poly(I:C) markedly slowed tumor growth and prolonged survival. This treatment was associated with a reduction in intratumoral CD11c+ dendritic cells and an influx of CD8+ T cells. Since intratumoral injection of plasmid DNA does not lead to efficient transgene expression, pSP-D-CD40L was also tested with cationic polymers that form DNA-containing nanoparticles which lead to enhanced intratumoral gene expression. Intratumoral injections of pSP-D-CD40L-containing nanoparticles formed from polyethylenimine (PEI) or C32 (a novel biodegradable poly(B-amino esters) polymer) in combination with CpG + poly(I:C) had dramatic antitumor effects and frequently cured mice of B16F10 tumors. These data confirm and extend previous reports that CD40 and TLR agonists are synergistic and demonstrate that this combination of immunostimulants can significantly suppress tumor growth in mice. In addition, the enhanced effectiveness of nanoparticle formulations of DNA encoding immunostimulatory molecules such as multimeric, soluble CD40L supports the further study of this technology for tumor immunotherapy.

## Introduction

A number of immunostimulants, such as anti-CTLA4 antibody [1], have been shown to be efficacious in treating established tumors in mice and several of these agents have advanced to clinical trials in humans. Recently, it has been shown that the local application of Toll-Like Receptor (TLR) agonists may have antitumor effects [2], [3], [4], [5]. For example, imiquimod cream (Aldera™) is effective for lentigo maligna (an in situ form of melanoma) [6] and basal cell carcinoma [7]. Equally promising in mice but difficult to apply in humans is the use of CD40 stimulation. Numerous studies have shown that agonistic antibody to CD40 can have major antitumor effects either on its own [8], [9], [10], [11], [12] or when combined with TLR agonists [13], [14], [15], [16]. However, agonistic anti-CD40 antibody can be toxic, especially if used repeatedly [12], [17]. CD40L itself has been used in several circumstances. As a single-trimer, soluble protein (sCD40LT, Avrend™, Immunex/Amgen), systemic therapy had significant antitumor effects, but also produced dose-limiting hepatic toxicity [18]. Efforts to deliver CD40L by injecting adenoviral vectors directly into tumors have shown promise [19], [20]. Alternatively, the co-delivery of CD40L along with defined tumor antigens may produce strong antitumor effects [21], [22].

From an immunological point of view, most of these immunostimulants activate dendritic cells from a resting, tolerogenic state to that of a fully effective antigen-presenting cell. In so doing, these immunostimulants counteract the deactivating effects of tumors on the dendritic cells in their immediate environment [23], [24], [25]. A recent insight is that DC activation is not a simple on/off switch but rather is a tunable pathway leading to qualitatively and quantitatively different outputs. For IL-12p70 production by DCs, for example, Napolitani et al. [26] found that the combination of two TLR agonists (e.g., poly(I:C) for TLR3 and R-848 for TLR7/8) was markedly synergistic, and the addition of CD40L led to a further 10- to 100-fold increase in IL-12p70 production. Zheng et al. showed the antitumor effects of CpG plus poly(I:C) stimulation of DC-tumor cell electrofusion hydrids [27]. Wells et al. have shown the antitumor effects of a combination of agonistic anti-CD40 antibody, CpG, poly(I:C), and IFN-γ delivered as a emulsion in squalene and Tween 80 [28]. These finding support earlier reports that CD40 stimulation combined with TLR agonists is capable of inducing strong antitumor CD8+ T cell responses [13], [14].

The present study was undertaken to examine the effects of combinations of TLR agonists along with a new form of CD40L that dramatically enhanced CD8+ T cell responses in a murine DNA vaccine model [29]. This form of CD40L was produced by fusing the extracellular domain of CD40L with the body of surfactant protein D (a spontaneously multimerizing molecule) resulting in a 4-trimer soluble protein encoded by the plasmid pSP-D-CD40L. In these previous studies, SP-D-CD40L led to enhanced CD40 activation and increased immune activation both in vitro and in murine vaccine models [29], [30]. In the present study, we used the B16F10 melanoma because it is a frequently studied, poorly immunogenic, spontaneously metastasizing tumor model that is very difficult to treat using immunotherapy [31]. Significantly, pSP-D-CD40L, CpG, and poly(I:C) showed activity when injected directly into established tumors every other day X 5. Synergy between these two TLR agonists and synergy between pSP-D-CD40L and TLR agonists were observed. In addition, enhanced delivery of pSP-D-CD40L in nanoparticles formed with cationic polymers increased the antitumor effects, indicating that DNA delivery into tumors is a surmountable barrier to this type of immunotherapy. Taken together, these studies show that DNA delivery of multimeric soluble CD40L is a practical means for providing CD40 stimulation in vivo.

## Materials and Methods

### Tumor Immunotherapy Plasmids

The construction of the CD40L and GITRL plasmids was previously described [29], [32]. The following murine plasmids were tested: pTr-CD40L, encoding 1-trimer soluble CD40L; pAcrp30-CD40L, encoding a 2-trimer soluble form of murine CD40L; pSP-D-CD40L, encoding a 4-trimer soluble form of murine CD40L; pSP-D-GITRL, encoding a 4-trimer soluble form of murine GITRL; and pcDNA3.1(+) (Invitrogen, Carlsbad, CA) empty vector as a control.

### Plasmid preparation

**Figure 1:**
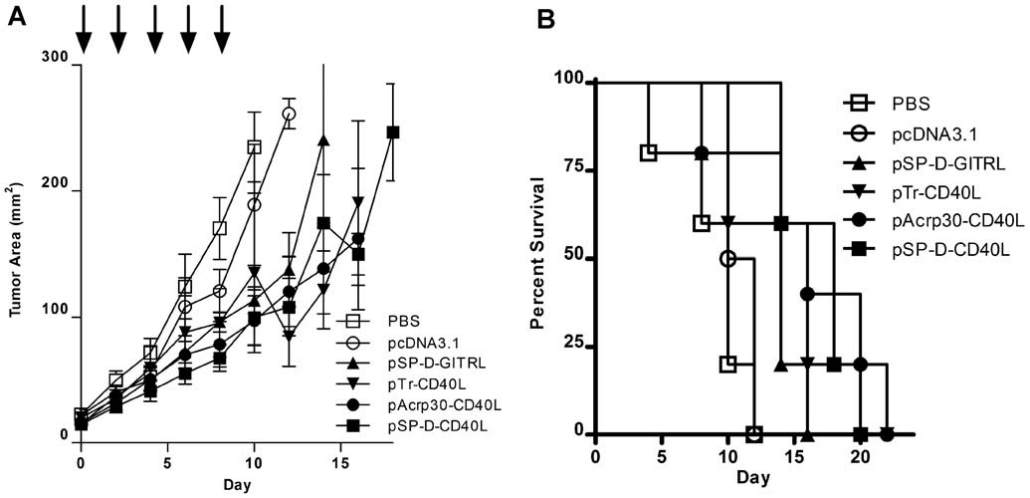
Antitumor effects of plasmids for multimeric, soluble CD40L and GITRL on established B16F10 melanoma tumors. B16F10 cells were injected s.c. in C57BL/6 mice. When the tumors were ≥4 mm in diameter, they were injected every other day X 5 with 50 µg of plasmid DNA. Three forms of CD40L were tested as fusion proteins (see text): 1-trimer soluble CD40L (pTr-CD40L); 2-trimer CD40L (pAcrp30-CD40L); and 4-trimer CD40L (pSP-D-CD40L). Additionally, a 4-trimer form of GITRL (pSP-D-GITRL) was tested for comparison. The negative control injections were either PBS or the empty expression plasmid pcDNA3.1. Panel A – Treatment with pSP-D-CD40L or pSP-D-GITRL slowed the growth of established B16F10 tumors. Each graph shows 5 mice per group for each treatment where day 0 indicates the time when the tumor became ≥4 mm and injections began and ending when fewer than 3 mice in each group remain alive. Injections continued every other day X 5, ending on day 8 (arrows). There was a significant reduction in tumor size (mean±SEM, n = 5) compared to control pcDNA3.1 or PBS using 2-trimer pAcrp30-CD40L, 4-trimer pSP-D-CD40L, and 4-trimer pSP-D-GITRL on day 8 as measured before the final injection (p<0.05 by Student's t test). Panel B – Treatment of established B16F10 tumors with pSP-D-CD40L significantly prolonged survival. While treatment with plasmids for all 3 forms of CD40L and 4-trimer GITRL showed a trend toward enhanced survival, this was only statistically significant for the 4-trimer pSP-D-CD40L plasmid (p<0.01 by log-rank test). Consequently pSP-D-CD40L was selected for further study.

Plasmids were propagated in *E. coli* strains XL1 blue or TOP10. Supercoiled plasmid DNA was isolated by anion-exchange chromatography resin (EndoFree Plasmid MaxiKit, QIAgen, Inc, Valencia, CA). Initial experiments indicated that the empty control vector pcDNA3.1 isolated by this method was capable of inducing an antitumor effect in mice, despite the fact that it was negative for endotoxin (<0.1 EU/ml) by Limulus Assay (QCL-1000, BioWhittaker, Walkersville, MD). Consequently, an additional purification step using Triton X-114 detergent extraction was used for the experiments shown in Figs. 1, 3–6 [33].

To prepare Triton X-114 detergent (Sigma, St. Louis, MO), it was pre-equilibrated by adding 10 volumes TE buffer (10 mM TRIS-HCl, 0.1 mM EDTA, pH 8.0), vortexed, incubated at 4°C for 6 hours, and then held at 37°C overnight. The later temperature is above the cloud point of Triton X-114, which then separates into a sublayer. At this point, the upper aqueous phase and any turbid material at the interface were removed and the detergent sublayer was harvested. This procedure was repeated a total of three times. The resulting buffer-equilibrated Triton X-114 was then stored below its cloud point at 4°C.

To use Triton X-114 after completing the EndoFree kit purification protocol above, plasmid DNA was suspended in endotoxin-free TE buffer (pH 8.0) at a concentration of 0.8 mg/ml. Endotoxin-free 3M sodium acetate, pH 5.2 (Sigma) was added to a final concentration of 0.3 M. Then a total of 0.03 volumes of pre-equilibrated Triton X-114 were added to the DNA solution (e.g., 30 µl per 1 ml) and the sample was vortexed thoroughly. After incubation below the Triton X-114 cloud point on ice for 15 minutes, the sample was heated to 37°C for 10 minutes to allow the two phases to separate, followed by centrifugation at 400 x g for 2 minutes at room temperature. The upper aqueous phase containing the DNA was then transferred to a new tube and another two cycles of extraction were performed for a total of three detergent extractions. Plasmid DNA in the final upper aqueous phase was precipitated by the addition of 0.7 volumes of room temperature isopropanol, followed by centrifugation at 13,000 rpm in a microcentrifuge for 10 minutes. The DNA pellet was then washed with cold 70% ethanol (endotoxin free), air-dried briefly, and dissolved in endotoxin-free TE buffer (pH 8.0) at 4°C for 1–2 days. The final plasmid concentrations were typically 5–7 mg/ml. The control pcDNA3.1 plasmid prepared in this manner had minimal antitumor effects, confirming the removal of an immunostimulatory contaminant in the initial plasmid DNA prepared using the EndoFree kit. Prior to injection into mice, plasmid DNA was diluted in Dulbecco's calcium- and magnesium-free phosphate buffered saline (PBS) to a concentration of 1 mg/ml (50 µg per 50 µl injection).

**Figure 2:**
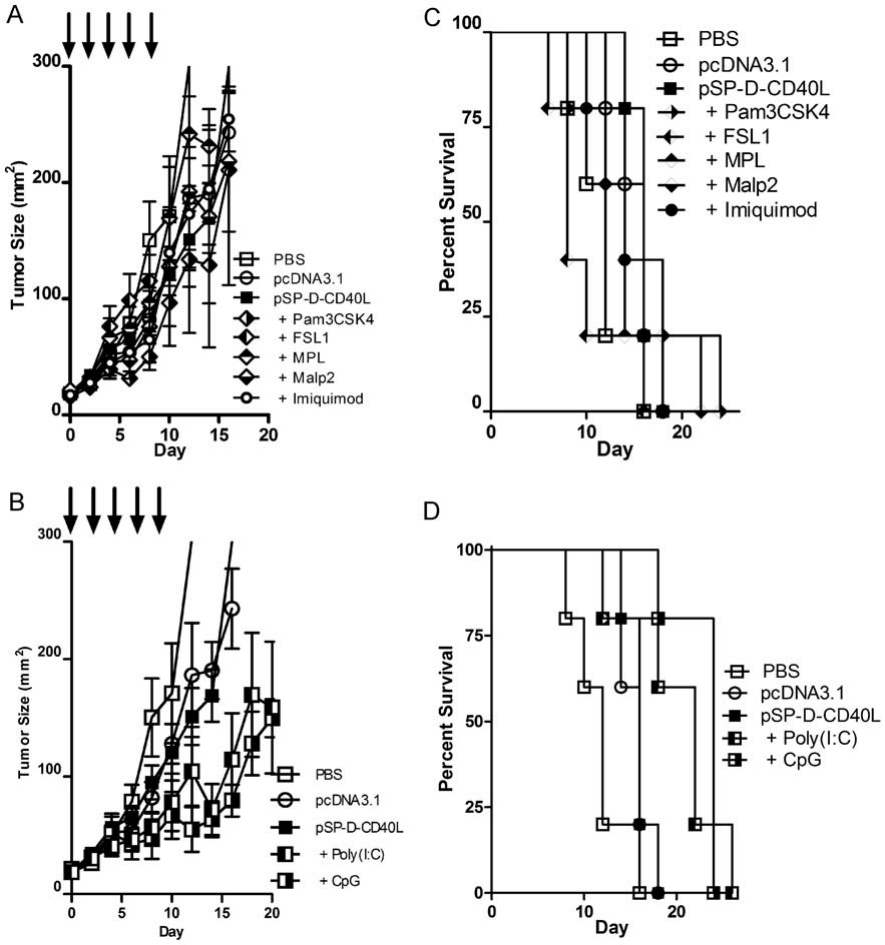
A screen of TLR agonists showed that CpG and poly(I:C) had additional antitumor effects when combined with 4-trimer CD40L plasmid DNA. Panels A and B – The combination of 4-trimer pSP-D-CD40L with CpG or poly(I:C), but not other TLR agonists tested, slowed the growth of established B16F10 tumors. As before, tumors that were ≥4 mm in diameter were injected with pSP-D-CD40L in combination with selected TLR agonists every other day X 5 (arrows). There were no apparent additive effects of Pam_3_CSK_4_ (TLR1/2), Malp2 (TLR2/6), FSL1 (TLR2/6), MPL (TLR4), and imiquimod (TLR7) (Panel A, mean±SEM, n = 5). The addition of poly(I:C) (TLR3) to pSP-D-CD40L showed a significantly stronger effect on tumor growth than pSP-D-CD40L alone from day 14 (Panel B, p<0.05 by Student's t test). CpG was clearly active when added to pSP-D-CD40L as compared to pSP-D-CD40L alone from day 14 (Panel B, p<0.01 by Student's t test). Panels C and D – The addition of CpG to pSP-D-CD40L resulted in a further survival benefit for mice with established B16F10 tumors. As expected from the tumor growth data, there was no increase in survival when Pam_3_CSK_4_, Malp2, FSL1, MPL, or imiquimod were added to pSP-D-CD40L treatment (Panel C). While the addition of poly(I:C) to pSP-D-CD40L showed a trend toward improved survival, this was not statistically significant when compared to pSP-D-CD40L alone (Panel D). In contrast, the addition of CpG to pSP-D-CD40L showed a clear survival benefit when compared to pSP-D-CD40L alone (Panel D, p<0.01 by log-rank test).

EndoFree DNA without Triton X-114 processing was used in the experiment in Fig. 2. As shown, pcDNA3.1 plasmid prepared in this manner slowed tumor growth but did not significantly prolong survival. Nevertheless, to avoid this potentially confounding effect, all other experiments were performed using EndoFree DNA processed by the Triton X-114 extraction protocol above. Also, whenever plasmid DNA was loaded into 0.5 ml insulin syringes, the 28G needle was first removed with pliers before drawing up the DNA in order to avoid shearing the supercoiled plasmids, following which the needle was reattached for injection into mice.

### Polymer DNA nanoparticles

Polyethylenimine (PEI) nanoparticles were made using In Vivo JetPEI™ and 10% glucose solution (Q-Biogene Inc., Montreal, Canada). First, the 10% glucose solution was diluted to 5% glucose using endotoxin-free sterile water for injection (Baxter, Deerfield, IL). Then the plasmid DNA stock in TE buffer (5–8 mg/ml) was diluted with 5% glucose solution to a final concentration of 1 µg DNA/µl volume. Separately, one part In Vivo JetPEI™ stock was diluted in 9 parts 5% glucose solution and mixed. Then, for each mouse injected, 50 µl of DNA solution was mixed with 50 µl of In Vivo JetPEI™ solution, pulse vortexed, quick spun in a microcentrifuge, and then allowed incubate at room for at least for 15 minutes prior to use. Tumors were injected with 100 µl of this mixture consisting of 50 µg plasmid DNA at a final concentration of 0.5 µg/µl in isotonic 5% glucose. This corresponds to an N/P ratio of 10∶1, i.e., 10 nitrogen residues of PEI per DNA phosphate.

Solid C32 polymer was synthesized at MIT and shipped on dry ice to San Diego. It was dissolved at 100 mg/ml in cell-culture grade sterile DMSO (Sigma-Aldrich, St. Louis, MO) at room temperature for 2 hours, following which the hygroscopic solution was aliquoted and stored at −80°C until use. C32 plasmid DNA nanoparticles were prepared shortly before use. The following steps yielded 500 µl of nanoparticle solution which is sufficient to inject 100 µl/tumor in 5 mice, and the actual amounts were scaled up proportionally according to the number of mice used. First, using a 1.5 ml microcentrifuge tube, plasmid DNA was added to sterile water for injection to make a final DNA concentration of 5 mg/ml in a volume of 50 µl. In a separate microcentrifuge tube, sterile water was used to prepare 25 mM sodium acetate from a commercial solution of 3 M sodium acetate, pH 5.2 (Sigma-Aldrich). Then 75 µl of the 25 mM sodium acetate solution was added to the tube containing 50 µl of DNA. Because the DNA was diluted from a stock dissolved in TE pH 8.0 (as opposed to DNA prepared in water as originally described by Anderson et al. [34]), there was a concern that the TRIS buffer could raise the pH in subsequent steps. Consequently, an additional 3 µl of 3 M sodium acetate, pH 5.2 was added to this 125 µl DNA-containing mixture. Then the tube was pulse vortexed to mix and quick spun to position the solution at the bottom of the tube. Second, in a separate microtube, the C32 solution was prepared at room temperature by combining 75 µl of cell-culture grade DMSO and 50 µl of the C32 stock solution (100 mg/ml in DMSO), pulse vortexed to mix, and then quick spun. Next, 125 µl of this C32 solution was added to the tube containing 125 µl of the DNA/sodium acetate solution, gently mixed by hand, and then quick spun. The resulting 250 µl solution was allowed to incubate at room temperature for 5 minutes. Lastly, 280 µl of calcium- and magnesium-free PBS was added to the 250 µl of C32 DNA mix, pulse vortexed, quick spun, and then 500 µl was loaded into a syringe for the injections (the extra 30 µl of PBS volume assured that a full 500 µl could be loaded into the syringe).

### TLR Agonists

TLR agonist compounds were prepared following their manufacturer's instructions. TLR1/2 agonist Pam_3_CSK_4_ (InvivoGen, San Diego, CA) was suspended at 1 mg/ml in PBS and administered at 5 µg per injection. TLR2/6 agonist FSL-1 (InvivoGen) was suspended at 0.2 mg/ml in PBS and administered at 1 µg per injection. The synthetic mycoplasmal lipoprotein and TLR2/6 ligand MALP2 (InvivoGen) was suspended in PBS at a concentration of 0.2 mg/ml and administered at 3 µg per injection. TLR3 agonist poly(I:C) (GE Amersham, Piscataway, NJ) was prepared by adding 20 ml PBS to the 50 mg in the vial, incubating at 60°C for 20 minutes, and then placing the vial in a beaker containing 100 ml water at room temperature to allow the RNA strands to slowly hybridize. Following this, the 2.5 mg/ml solution was aliquoted and stored at −80°C. Just prior to use, the poly(I:C) stock was diluted 1∶5 in PBS to provide a 500 µg/ml working solution of which 25 µg was administered per injection. TLR4 agonist monophosphoryl lipid A (MPL), a detoxified form of lipid A (Avanti Polar Lipids, Alabaster, AL), was dissolved in 50% ethanol at 1 mg/ml and administered at 10 µg per injection. TLR7 agonist imiquimod acetate (Sequoia Research Products, Oxford, UK) was suspended in particulate form in a mixture of 1.5% Methylcellulose and 0.5% Tween 80 [35] at a concentration of 10 mg/ml and administered at 25 µg per injection. TLR9 agonist CpG 1018 5′-TGACTGTGAACGTTCGAGATGA-3′ (all DNA linkages phosphorothioate), a B Class CpG, was a gift of Dr. Eyal Raz [36] or was purchased from Trilink Biotechnologies (San Diego, CA) as a reverse phase-HPLC purified product. The lyophilized CpG 1018 powder was resuspended in water at a concentration of 5 mg/ml and stored at −80°C until use. Just prior to use, the CpG stock was diluted 1∶5 in PBS to provide a 500 µg/ml working solution of which 25 µg was administered per injection. With the known exception of MPL, all solutions were endotoxin-free. The volume of the TLR agonist injections was held constant at 50 µl, using PBS as necessary to adjust the total amount to this volume.

### Tumor Cell Lines

B16F10 melanoma from C57BL/6 mice [37] was obtained from the American Type Culture Collection (ATCC), Manassas, MD. Cells were cultured in RPMI 1640, 2 mM L-glutamine, and 10% FBS (HyClone, Thermo Fisher Scientific, Waltham, MA), and were negative for mycoplasma and other adventitious agents by PCR testing (IMPACT II, RADIL, University of Missouri).

### Tumor Initiation and Immunotherapy

Mice were studied under a protocol approved by the Institutional Animal Care and Use Committee of the VA San Diego Healthcare System, and the “rinciples of laboratory animal care”(NIH publication No. 85–23, revised 1985) were followed. Cultured tumor cells were detached from flasks using trypsin/EDTA followed by neutralization with cold RPMI +10% FBS and pelleting at 300 x g for 6 minutes. The cells were then washed 3 times with cold PBS and resuspended at 5×10^6^ cells/ml in PBS. To initiate tumors, a total of 5×10^5^ cells (0.1 ml) were injected subcutaneously into the abdomen of 6–8 week old female C57BL/6 mice (The Jackson Laboratory, Bar Harbor, ME). When the s.c. tumors became palpable and measured ≥4 mm in diameter (determined as the mean of two orthogonal measurements), the mice were eartagged and considered to be at day 0 of the treatment protocol. Prior to performing the injections into tumors, mice were first lightly anesthetized with isoflurane gas. Then a total of 100 µl of DNA solution or 50 µl of TLR agonists were injected into or around the tumor using a 0.5 ml insulin syringe with a 28 gauge needle. When combinations of plasmid DNA and TLR agonists were used, the DNA was injected on days 0, 2, 4, 6, and 8, and the TLR agonists were injected the next day on days 1, 3, 5, 7, and 9. This schedule was based on a report that dendritic cells are best stimulated when TLR stimulation follows CD40 stimulation rather than the reverse or simultaneously [38]. For all treatments, peritumoral injections were repeated every other day for a total of 5 plasmid DNA injections. The tumor diameter in two orthogonal dimensions was measured with an electronic caliper beginning on the day of initial injection and every other day until the endpoint was reached. Mice were euthanized when tumors became ≥15 mm in mean diameter or ulcerated. Survival was calculated as the number of days from the first injection on day 0 when the tumors were ≥4 mm in diameter until the mice were either found dead or required euthanasia.

### Tumor Histology

Tumor cells were injected s.c. (5×10^5^ per mouse) on the abdomen and allowed to grow to at least 7 mm in diameter. Mice were then given peritumoral injections of immunostimulatory compounds every other day X 5 (as detailed above) and then euthanized two days later on day 10 by pentobarbital injection. The tumors were excised and divided so that one half was fixed in 1% paraformaldehyde and embedded in paraffin, and the other half was flash frozen in OCT compound (Tissue-Tek, Thermo Fisher Scientific). Serial sections (10 µm in thickness) were obtained from the paraffin-embedded tissue and processed for hematoxylin and eosin staining. For immunofluoresence microscopy, the OCT-embedded tissue was cut into 10 µm serial sections with a cryostat and stained by standard antibody methods. The following antibodies were used: fluorescein-conjugated CD11c clone HL3 (BD Pharmingen, San Diego, CA); phycoerythrin (PE)-conjugated CD8 clone 53–6.7 (BD Pharmingen); and biotinylated F4/80 clone BM8 (eBioscience, San Diego, CA) which was used with PE-conjugated streptavidin (BD Pharmingen). Slides were examined with a Zeiss Axioskop microscope and images were recorded using an Optronics CCD camera. Duplicate tumors were evaluated for each condition.

### Statistics

In order to compare tumor growth, the geometric mean tumor diameters of surviving mice were compared on the days stated using Student's t test (Prism 4.0 Software, GraphPad Systems, San Diego, CA). To compare Kaplan-Meier survival plots, a log-rank test (Mantel-Haenszel method) was used to determine the significance of the differences in survival between groups. A p value of <0.05 was considered significant.

## Results

### Characteristics of the B16F10 melanoma model in C57BL/6 mice

We deliberately chose B16F10 melanoma as a treatment-resistant tumor model that could be used to discriminate between strong immunotherapy regimens. B16F10 is ideal for this purpose because it is (1) poorly immunogenic, (2) lacks foreign antigens, (3) fast growing, (4) highly metastatic, and (5) rapidly fatal. (1) B16F10 is poorly immunogenic. Of all of the B16 sublines tested, B16F10 has the lowest level of surface MHC Class I and almost unmeasurable amounts of antigen processing machinery components (TAP1, LMP2, LMP7, LMP10, PA28α, and PA28β), although these proteins could be significantly upregulated by treatment with interferon-γ [39]. (2) B16F10 lacks foreign antigens that are likely to be recognized by the immune system. Instead, its principal rejection antigen is tyrosinase-related protein 2 (TRP2), a self-antigen that tends to elicit low affinity, poorly functional CD8+ T cell responses [40]. In contrast, some studies use B16F10 transfected with chicken ovalbumin, a foreign antigen that is sometimes maintained in cells using the *neo* gene for G418 selection which is itself a strong antigen for immune rejection [41]. Even green fluorescent protein (GFP) is a foreign antigen in mice [42]. The presence of such foreign antigens can artifactually increase the antigenicity of otherwise poorly immunogenic tumor cells and thereby confound the results of an immunotherapy study. (3) B16F10 is fast growing compared with many tumors commonly studied. As a result, B16F10 is harder to treat than tumors formed from either parental B16 cells or more slowly growing variants of this tumor cell line such as B16-BL6. For example, B16-BL6 was used in the original description of therapeutic vaccination with irradiated GM-CSF-transfected tumor cells and blocking antibody to CTLA-4. In that study, 10^4^ B16-BL6 tumor cells were injected to initiate a tumor and the treatment had to be given by day 4 postinjection at which time the tumors were barely measurable [43]. In the present study, in contrast, tumors were initiated by injecting 50X more cells (5×10^5^ rapidly growing B16F10 cells) and treatment was delayed for 5–7 days until the tumors were ≥4 mm in mean diameter. (4) B16F10 is highly metastatic by design. Fidler originally derived the B16F10 subline by injecting parental B16 cells intravenously, harvesting tumor cells from a lung metastasis, and then repeating this procedure for a total of 10 cycles [44]. (5) B16F10 is rapidly fatal, generally within 10 days of s.c. injection under the conditions employed here. To be effective, this means that an antitumor immunotherapy must induce strong immunity very quickly in order to overtake the tumor's rapid growth.

### For established B16F10 melanoma, local treatment with plasmid DNA for multimeric soluble CD40L, pSP-D-CD40L, slowed tumor growth and prolonged survival

Previous studies have shown that CD40L is most effective when it clusters its receptor, CD40, on the membranes of responding cells [45], [46]. In our previous study [29], we used the pcDNA3.1 expression vector to prepare plasmids encoding soluble CD40L with varying degrees of valency by fusing the extracellular domain (ECD) of CD40L with multimerization scaffolds selected from other proteins. A plasmid for 1-trimer soluble CD40L, pTr-CD40L, was produced by fusing an N-terminal isoleucine zipper to the CD40L ECD, similar to the widely studied sCD40LT (Avrend™ Immunex/Amgen) [47]. A plasmid for 2-trimer CD40L, pAcrp30-CD40L, was produced by fusing the body of Acrp30 (a V-shaped molecule with two trimeric arms) with the CD40L ECD [48]. A plasmid for 4-trimer CD40L, pSP-D-CD40L, was produced by fusing the body of surfactant protein D (an X-shaped molecule with four trimeric arms) with the CD40L ECD. In the previous DNA vaccine study [29], the adjuvant activity of soluble CD40L was directly proportional to the number of trimers per molecule (4>2>1), with pSP-D-CD40L being most active.

To determine if soluble CD40L multivalency was also important in the tumor immunotherapy setting, established B16F10 tumors were injected peritumorally with PBS alone, control empty expression plasmid pcDNA3.1, or plasmids for the three soluble forms of CD40L: 1-trimer pTr-CD40L; 2-trimer pAcrp30-CD40L; and 4-trimer pSP-D-CD40L. An alternative TNF superfamily (TNFSF) molecule, GITRL, was also tested as a 4-trimer soluble protein construct (pSP-D-GITRL) [29].

As shown in Fig. 1A, tumor growth was significantly slowed after peritumoral injections of plasmids for 4-trimer CD40L (pSP-D-CD40L) as compared to empty vector (beginning on day 8 tumor size measurements differed at the p<0.05 level by Student's t test). If PBS was used for comparison, then 2-trimer CD40L (pAcrp30-CD40L) and 4-trimer GITRL (pSP-D-GITRL) also appeared to slow tumor growth from day 8 (p<0.05). However, PBS is an unsatisfactory control because DNA itself may have mild immunostimulatory effects [49], [50]. As shown in Fig. 1B, there was a trend toward prolonged survival with all of the TNFSF molecules, but statistical significance with this small number of animals (n = 5/group) was only reached using 4-trimer pSP-D-CD40L (p<0.01, log-rank test compared with PBS or pcDNA3.1). For this reason, the 4-trimer version of soluble CD40L (pSP-D-CD40L) was selected for further studies.

### For established B16F10 melanoma, pSP-D-CD40L combined with either CpG or poly(I:C) resulted in stronger antitumor effects than pSP-D-CD40L alone

Both in vitro [26], [51], [52], [53] and in vivo studies [13], [14], [54], [55] have noted a more than additive effect of combining agonistic anti-CD40 antibody with TLR agonists such as CpG oligonucleotide, poly(I:C) and derivatives of the TLR7 agonist imiquimod. However, due to the potential toxicity of agonistic anti-CD40 antibody treatment [17], the application of agonistic anti-CD40 antibodies to humans may be problematic [8]. In contrast, we observed no toxic effects when pSP-D-CD40L was used as an adjuvant in the prior DNA vaccine study [29]. Consequently, synergistic interactions between pSP-D-CD40L and TLR agonists were sought. Established B16F10 tumors were treated with a combination of pSP-D-CD40L (50 µg injected peritumorally every other day X 5) with or without selected TLR agonist compounds, prepared and dosed as described in Methods. As shown in Figs. 2A and 2B, most of the TLR agonists tested failed to improve upon the antitumor effects of pSP-D-CD40L alone. We cannot rule out that many of these TLR agonists would have been more effective if they had been formulated or administered differently. In contrast, the combination of pSP-D-CD40L + CpG or pSP-D-CD40L + poly(I:C) significantly inhibited tumor growth (Fig. 2C, p<0.01 and p<0.05 respectively from day 14 by Student's t test). The finding that poly(I:C) synergized with pSP-D-CD40L is similar to the report of Liu et al. that poly(I:C) combined with agonistic CD40 antibody protected mice from J558 plasmacytoma tumors [14]. There was a trend toward a further survival advantage when CpG or poly(I:C) was added to pSP-D-CD40L, but this difference was only statistically significant with CpG using this small number of animals (Fig. 2D, p<0.01 by log-rank test comparing pSP-D-CD40L with pSP-D-CD40L + CpG, n = 5/group).

### For established B16F10 melanoma, pSP-D-CD40L combined with two active TLR agonists resulted in increased antitumor activity

**Figure 3:**
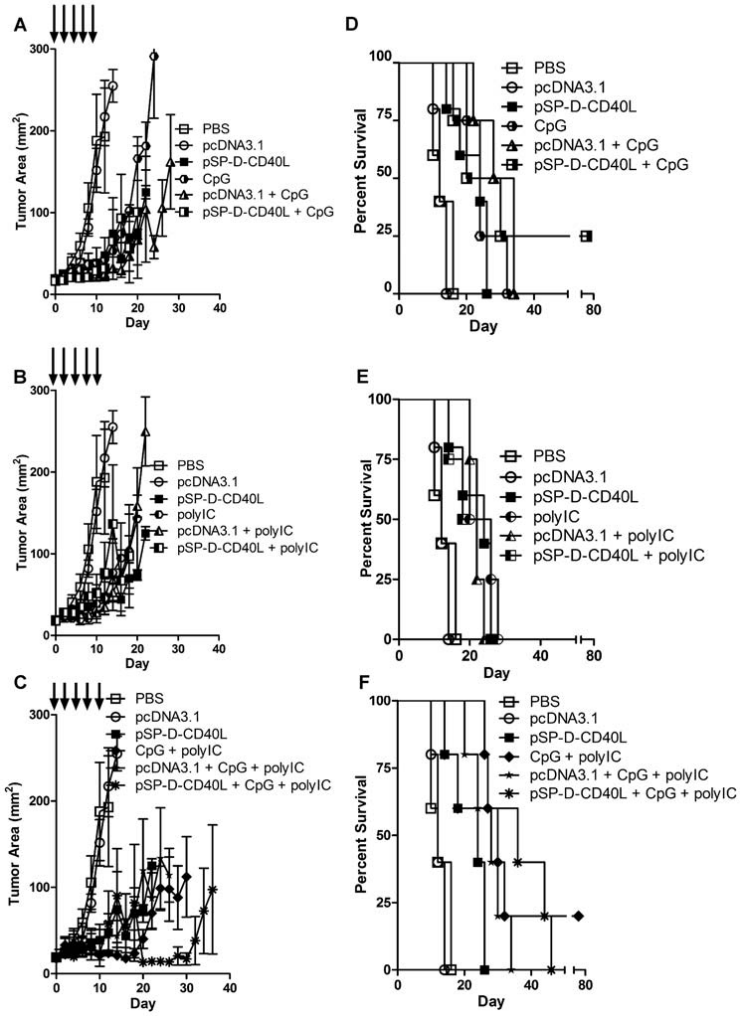
Combinations of pSP-D-CD40L, CpG, and poly(I:C) showed strong antitumor effects on established B16F10 melanoma. Given the promising data of Fig. 2, further studies were done to determine the relative contributions of pSP-D-CD40L, CpG, and poly(I:C) and the effects of using them in a triple combination. Twelve groups of mice (5/group) were studied in parallel. For display purposes, the data are grouped into three rows of graphs focusing on CpG (top row), poly(I:C) (middle row), and CpG + poly(I:C) (bottom row). Panels A, B, and C – While each individual agent slowed tumor growth, the most significant antitumor effect was produced by the combination of pSP-D-CD40L + CpG + poly(I:C). Panel A shows that CpG alone significantly slowed tumor growth compared to either PBS or pcDNA3.1 alone from day 12 (p<0.01 by Student's t test, mean±SEM, n = 5). In this fully controlled experiment, however, it was clear that the addition of pSP-D-CD40L to CpG produced no further antitumor effects (p>0.05). Similarly, Panel B shows that poly(I:C) alone significantly slowed tumor growth when compared to PBS or pcDNA3.1 alone from day 12 (p<0.01). Again, however, the combination of pSP-D-CD40L + poly(I:C) produced no further antitumor effects (p>0.05). Interestingly, as shown in Panel C, the double combination of CpG + poly(I:C) significantly reduced tumor growth beyond that produced by CpG alone (p<0.05 on day 24 on the combination as compared to CpG alone). The addition of pSP-D-CD40L to the two TLR agonists, CpG and poly(I:C), produced an even stronger antitumor effect (Panel C, p<0.05 on day 24 comparing the triple combination to CpG + poly(I:C)). Panels D, E, and F – For survival, the addition of pSP-D-CD40L did not increase the antitumor effects seen with CpG alone. All three agents (pSP-D-CD40L, CpG, and poly(I:C)) improved survival as single therapies. From pairwise comparisons, the survival benefit was greatest with CpG and less prominent with pSP-D-CD40L and poly(I:C). The combination of CpG + poly(I:C) improved survival further compared to poly(I:C) alone (p<0.05 by log-rank test). Although the effects on tumor growth indicated that the double combination of TLR agonists CpG + poly(I:C) was better than each alone, this was not reflected in the survival data. Similarly, the superiority of the triple combination of pSP-D-CD40L + CpG + poly(I:C) seen in the tumor growth studies was not statistically significant from the survival data.

Napolitani et al. reported that the triple combination of cells bearing membrane CD40L plus two different TLR agonists (LPS + poly(I:C), LPS + R848, or poly(I:C) + R848) could produce even stronger DC stimulation than that seen with CD40L and a single TLR agonist [26]. It was suggested that the combination of a MyD88 pathway agonist with a TRIF pathway agonist was important for the TLR synergy observed [26], [56]. To test this possibility in the B16F10 tumor system, various combinations of pSP-D-CD40L, CpG, and poly(I:C) were injected into established B16F10 tumors every other day X 5 (Fig. 3). All injections containing one or more of the three immunostimulants inhibited tumor growth (Figs. 3A, 3B, and 3C, p<0.01 from day 12 by Student's t test compared with pcDNA3.1). Importantly, the triple combination of pSP-D-CD40L + CpG + poly(I:C) was significantly better at slowing tumor growth when compared to the double combination of CpG + poly(I:C) without pSP-D-CD40L (substituting pcDNA3.1 instead, Fig. 3C, p<0.05 from day 24 by Student's t test). Similarly, a significant survival advantage was seen with all compound combinations compared to pcDNA3.1 empty plasmid vector (Fig. 3D, 3E, and 3F, p<0.01 by log-rank test). Additionally, the triple combination showed a trend to improved survival beyond that induced by any two combinations of pSP-D-CD40L, CpG or poly(I:C), although this did not reach statistical significance in the small number of animals used in this study (Fig. 3F, n = 5/group). Interestingly, in the mice cured of B16F10 melanoma in 11 experiments, there was no autoimmune loss of pigmentation (vitiligo) even after four months of observation.

### Mice cured of tumors were resistant to rechallenge with the same tumor type

There are many possible antitumor mechanisms that could account for the effects shown in the previous experiments. To determine if immunological memory was generated during tumor eradication, mice cured of B16F10 for greater than 90 days were resistant to rechallenge with the homologous tumor cell line (data not shown). In a related study of AB1 mesothelioma treatment with pSP-D-CD40L and TLR agonists, the cured mice were resistant to homologous AB1 rechallenge but not to a heterologous A20 tumor challenge [57]. Taken together, these data indicate that a specific memory response was present after tumor cure and argue against a long lasting non-specific increase in non-immune antitumor mechanisms.

### CD8 T cell infiltration is associated with successful tumor immunotherapy

**Figure 4:**
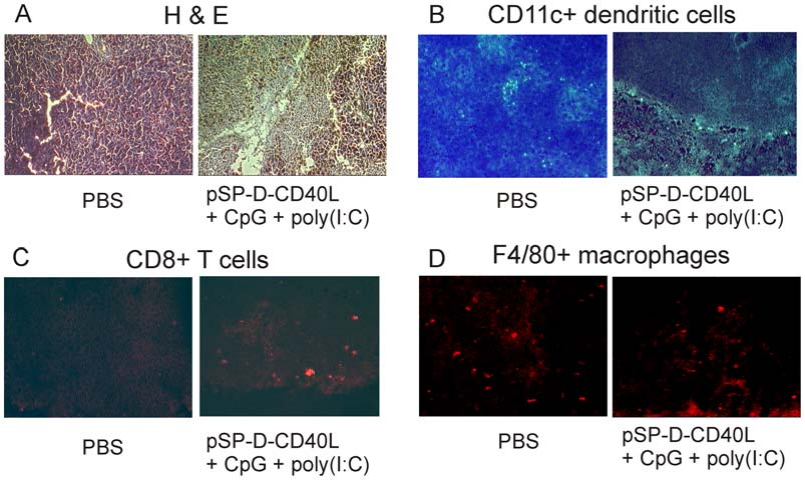
Tumor-dependent differences in the immunohistology of induced tumor regression. Panel A – Histology of control and treated tumors. Tumors were injected every other day X 5 with PBS as a control or with the triple combination of pSP-D-CD40L + CpG + poly(I:C). As shown in Figure 3, the triple combination slowed the growth of tumors, and occasionally led to tumor eradication. Two days after the last injection, tumor tissue was processed for histology by staining with hematoxylin and eosin. Tumors treated with PBS showed areas of spontaneous necrosis suggesting that the rapidly growing tumor cells often outgrow their blood supply. After treatment with the triple combination, large areas of necrotic tissue appeared containing fragmented cells and nuclear remnants consistent with a cell death process that exceeded the availability of phagocytic macrophages to clear the debris (see Panel D). Panel B – CD11c antibody staining for dendritic cells. B16F10 tumors injected with PBS as a control contained identifiable CD11c+ dendritic cells. After treatment with the triple combination, even fewer dendritic cells were found in the tumors. Panel C – CD8 antibody staining. For tumors injected with PBS as a control, relatively few CD8+ T cells were seen. However, following injections with the triple combination, there was a marked increase in intratumoral CD8+ T cells in all tumor sections examined. Panel D – F4/80 antibody staining for macrophages. Tumors injected with PBS as a control contained relatively few F4/80+ macrophages and there was no appreciable increase in F4/80+ macrophages following treatment with the triple combination.

Given the possible role of adaptive immunity in tumor eradication, tumor sections were analyzed for the presence or absence of immune cells, including DCs, CD8+ T cells, and macrophages. B16F10 tumors were treated with pSP-D-CD40L + CpG + poly(I:C) five times as detailed in Materials and Methods, followed by tumor dissection two days later. At this time point, the tumors treated with pSP-D-CD40L + CpG + poly(I:C) had an increased number of necrotic areas compared to PBS treated tumors, as shown by hematoxylin and eosin staining (Fig. 4A). These cells were identified as necrotic because there was no significant increase in apoptosis compared to baseline as detected by TUNEL staining (data not shown).

To identify immune cells within the tumors, frozen sections were stained for the presence of CD11c+ DCs, CD8+ T cells, and F4/80+ macrophages (Figs. 4B–4D). Tumors treated with pSP-D-CD40L + CpG + poly(I:C) showed a decrease in intratumoral dendritic cells (Fig. 4B). This may have resulted from the activation of these cells followed by their migration to the tumor-draining lymph nodes (TDLNs) [58]. Consistent with this interpretation, there was an influx of CD8+ T cells (Fig. 4C), suggesting a role for CD8+ T cell mediated activity in the anti-tumor activity observed. In a related study of AB1 mesothelioma, a dense tumor for which pSP-D-CD40L injections were not very effective, we observed a large macrophage infiltration [57]. CD40-activated macrophages could potentially mediate tumor cell death by expressing TRAIL, although no evidence for TRAIL was found in a study of B16 melanoma treated with agonistic anti-CD40 antibody plus CpG [59]. However, very little macrophage influx was seen in the present study of B16F10 tumors (Fig. 4D), suggesting fine differences in the mode of tumor eradication that could be related to differences in the microenvironment of these different tumor types.

### Enhanced pSP-D-CD40L DNA delivery using polyethylenimine (PEI) or C32 nanoparticles combined with CpG and poly(I:C) led to very strong antitumor effects and long-term tumor-free survival

As previously shown by Anderson et al., intratumoral injection of “naked” DNA is a very inefficient way to express a plasmid transgene in tumors. However, intratumoral injection of nanoparticles formed from plasmid DNA and cationic polymers such as PEI is dramatically better at producing transgene expression. C32, a novel poly(beta-amino esters) cationic polymer, is even more effective than PEI for intratumoral injection [34]. Consequently, these DNA delivery agents were studied in the B16F10 tumor model.

**Figure 5:**
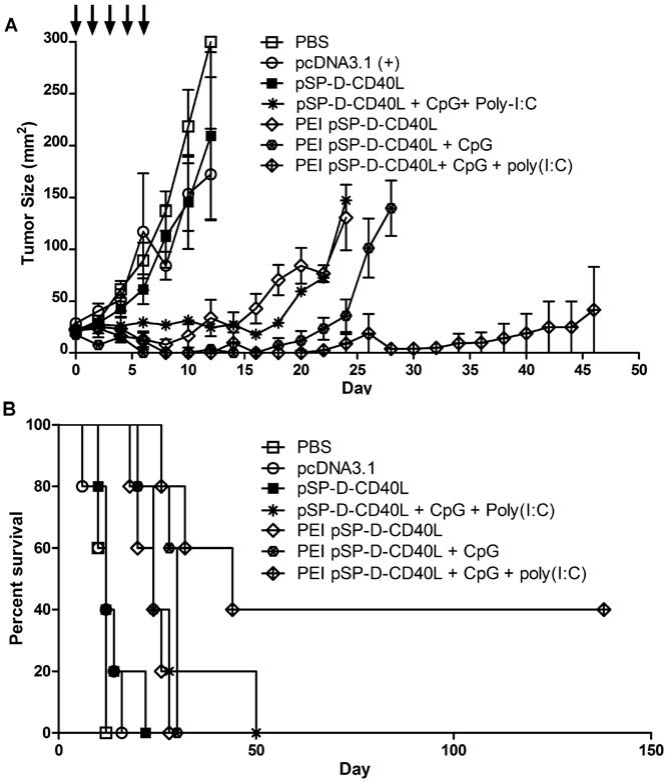
PEI nanoparticle delivery of pSP-D-CD40L slowed tumor growth and prolonged survival. The data shown are representative of three independent experiments. Panel A – Antitumor effects of PEI plasmid DNA nanoparticles prepared with pSP-D-CD40L alone or in combination with CpG or CpG + poly(I:C). The role of DNA transfection efficiency was tested by preparing nanoparticles formed from PEI and pSP-D-CD40L plasmid DNA. Intratumoral injections of PEI pSP-D-CD40L nanoparticles led to significantly slower tumor growth (p<0.05 on day 10) when compared to the injection of naked pSP-D-CD40L plasmid alone. Panel B – Survival benefit of PEI pSP-D-CD40L nanoparticle injections in combination with CpG + poly(I:C). As expected from the tumor growth data, pSP-D-CD40L formulated with PEI was able to enhance mouse survival when combined with CpG and poly(I:C) TLR agonists. This combination therapy resulted in long-term-tumor free survival of 2/5 mice (p<0.01 compared to pcDNA3.1)).

In Vivo JetPEI™ is a commercial preparation of polyethylenimine that has been optimized for in vivo transfection [60]. In Fig. 5, nanoparticles formed using JetPEI™ and plasmid DNA were compared with injections of plasmid DNA alone. As before, intratumoral injections of pSP-D-CD40L alone slowed tumor growth (Fig. 5A) and prolonged survival (Fig. 5B), and the antitumor effect was markedly augmented by combination with CpG and especially CpG + poly(I:C). Remarkably, however, pSP-D-CD40L delivered as a PEI nanoparticle had dramatically improved antitumor activity. PEI pSP-D-CD40L nanoparticles controlled tumor growth nearly as well as the triple combination of pSP-D-CD40L naked DNA plus CpG and poly(I:C). Even more striking, PEI pSP-D-CD40L nanoparticles combined with CpG + poly(I:C) strongly reduced B16F10 tumor growth and lead to long-term, tumor-free survival of ∼40% of mice in repeated experiments. These cured mice remained tumor-free for over a year, did not have vitiligo or other signs of autoimmunity, and resisted re-challenge with B16F10 melanoma.

While these results with PEI pSP-D-CD40L nanoparticles were exciting, other experiments indicated that PEI nanoparticles made with pcDNA3.1 control plasmid DNA also had low-level antitumor activity. Furthermore, scarring was observed at the site of PEI nanoparticle injection, confirming the cytotoxicity of PEI previously reported by others [61]. Given these limitations, we also tested C32, a novel poly(beta-amino esters) polymer that was selected from a polymer library based on its superior in vitro transfection activity. More importantly, as shown by Anderson et al., gene expression form intratumoral injections of C32 plasmid DNA nanoparticles was 4-fold stronger than with PEI nanoparticles and 26-fold stronger than intratumoral injection with naked DNA. Also, unlike PEI, C32 is nontoxic to cells in vitro [34].

**Figure 6:**
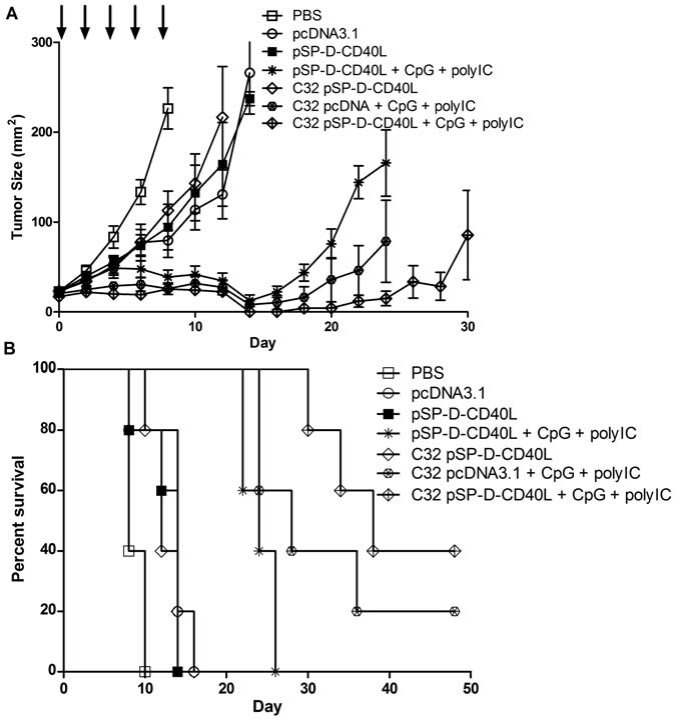
C32 nanoparticle delivery of pSP-D-CD40L slowed tumor growth and prolonged survival. The data shown are representative of three independent experiments. Panel A – Antitumor effects of C32 nanoparticles prepared with pSP-D-CD40L plasmid vs. control pcDNA3.1 plasmid either alone or in combination with CpG or CpG + poly(I:C). The role of DNA transfection efficiency was tested by preparing nanoparticles formed from C32 and pSP-D-CD40L or C32 and control pcDNA3.1 plasmid DNA. Intratumoral injections of C32 pSP-D-CD40L nanoparticles plus CpG + poly(I:C) led to significantly slower tumor growth when compared to the injection of naked pSP-D-CD40L plasmid + CpG + poly(I:C) (p<0.01 on day 24). Panel B – Survival benefits of C32 pSP-D-CD40L nanoparticle injections in combination with CpG + poly(I:C). As expected from the tumor growth data, injections of nanoparticles formulated with C32 and pSP-D-CD40L enhanced survival when combined with CpG + poly(I:C) TLR agonists. Although this survival benefit was not significantly better than a similar combination using pcDNA3.1 nanoparticles instead of pSP-D-CD40L nanoparticles (p>0.05), it was significantly better than pSP-D-CD40L naked DNA plus CpG + poly(I:C) (p<0.01).

As shown in Fig. 6, C32 nanoparticles also enhanced the antitumor effects of pSP-D-CD40L when combined with CpG + poly(I:C). In this case, C32 pSP-D-CD40L nanoparticles were not significantly better than that of pSP-D-CD40L naked DNA either in terms of tumor growth (Fig. 6A) or survival (Fig. 6B), a reproducible finding in three experiments. However, combining C32 pcDNA3.1 nanoparticles with CpG + poly(I:C) led to very significant antitumor effects. Indeed, C32 pcDNA3.1 nanoparticles combined with CpG + poly(I:C) was nearly as effective as pSP-D-CD40L naked DNA combined with CpG + poly(I:C) both in terms of tumor growth (Fig. 6A) and prolongation of survival (Fig. 6B). However, consistently superior antitumor activity was found using C32 pSP-D-CD40L nanoparticles combined with CpG + poly(I:C), indicating that C32 enhanced the effects of pSP-D-CD40L when combined with CpG + poly(I:C). From these data, we conclude that the C32 nanoparticle system may have an important role in advanced tumor immunotherapies based on pSP-D-CD40L in combination with TLR agonists.

## Discussion

These studies were undertaken to test the hypothesis that immune activators could induce significant antitumor effects in immunocompetent mice without using added tumor antigens. Two categories of agents were selected for study: CD40L and TLR agonists. Numerous studies have shown that strong CD40 stimulation can lead to the eradication of established tumors [8], [9], [10], [12], [20], [62]. Particularly noteworthy was the demonstration by van Mierlo et al. that agonistic anti-CD40 antibody leads to the rejection of tumors formed by malignant cells that themselves lack the CD40 receptor, indicating that a primary effect of this treatment is on the host immune response and not necessarily on the tumor cells themselves [12]. Expanding upon this concept, Hanks et al. constructed transgenic mice with DCs expressing a CD11c promoter-driven CD40 intracytoplasmic construct that could be multimerized by the addition of a cell-permeable chemical crosslinker. In the TRAMP-C model of spontaneous prostate tumor formation, the simple addition of the chemical crosslinker that only activated CD40 downstream pathways in dendritic cells was sufficient to lead to tumor eradication [62]. One potential mechanism of CD40 antitumor activity is through Th17 cells. CD40 stimulation has been shown to generate IL-23 and IL-6 which promote the activity of Th17 cells and can lead to anti-B16-F10 activity [63], [64]. Also, when CD8+ T cells are elicited by CD40-stimulated DCs, these cells do not express the negatively acting PD-1 surface protein, in contrast to other methods of DC activation [65]. These studies underscore the potential significance of CD40 stimulation as the foundation of an antitumor immunotherapy, and also prove the requirement for CD40 multimerization to provide optimal DC activation.

A number of studies have shown that many effects of CD40 stimulation are significantly magnified by an additional TLR stimulus. For example, CD40 stimulation alone induced DCs to produce only modest levels of IL-12p70, whereas the addition of TLR agonists to CD40 stimulation resulted in very high levels of IL-12p70 production [26], [53], [66]. Consistent with this CD40/TLR synergy in vitro, Ahonen et al. showed that CD40 stimulation combined with various TLR agonists could be used to generate exceptionally strong CD8 responses in vaccinated mice [13]. Their more recent studies showed that tumor antigen plus agonistic anti-CD40 antibody combined with a TLR7 agonist reduced B16F10 lung metastases in mice, though the treatment began only four days after the intravenous injection of tumor cells, rather than being tested on established tumors [67]. To understand the mechanism of combined therapy, Zhu et al. established a role for MyD88-dependent and independent pathways in the interplay between different TLR agonists [68], a hypothesis that could also be applied to MyD88-independent, CD40-mediated stimulation. Taken together, these studies provided the rationale for combining CD40L with TLR agonists in the present study.

A principal result was that pSP-D-CD40L has antitumor activity when injected directly into the tumor bed. This route of injection was modeled after numerous studies showing that peritumoral injections of radioactive tracers quickly localize to the tumor draining lymph nodes (TDLNs), a technique that is in wide use for “sentinel node biopsies” in breast cancer surgery. There is a great deal of evidence that TDLNs contain dendritic cells already charged with tumor antigens [14], [69], [70], [71], [72]. In this case, the protein expressed from pSP-D-CD40L injected into the tumor bed would either be carried by lymphatics to the TDLNs, or the plasmid DNA itself could travel to the TDLNs for uptake and expression by DCs there. In either case, this is a very inefficient way to introduce CD40L into a host. Studies by Anderson et al. using a luciferase plasmid have shown that direct intratumoral injections of naked DNA led to very little gene expression but that this could be greatly augmented by nanoparticle-mediated DNA delivery [34]. Consequently, the demonstration here that pSP-D-CD40L as naked DNA slowed the growth of B16F10 melanoma likely underestimates the true potential of this molecule. As shown by the studies with PEI (Fig. 5) and C32 (Fig. 6) nanoparticles, improved methods of DNA delivery are essential to define the full potential of pSP-D-CD40L for tumor immunotherapy.

Given this caveat, it is remarkable that CD40L/TLR agonist synergies were detected. For B16F10 melanoma, the antitumor effect of combined CpG + poly(I:C) was enhanced by the addition of pSP-D-CD40L naked DNA (Fig. 3A). Another result of this study was the effectiveness of TLR agonists as antitumor agents, as previously described in other reports. In an initial screen for synergy with pSP-D-CD40L, several agents showed no demonstrable effects (Pam_3_CSK_4_, Malp2, FSL1, MPL, imiquimod). These are hydrophobic compounds whose formulation may not have been optimal for this mode of delivery. Nevertheless, this schedule of repeated administration of TLR agonists is in line with the results of Yang et al. who found that TLR agonists could induce antitumor effects but only if they were repeatedly administered [5]. However, the limitation to 5 treatments over an 8 day period described in the present study was somewhat arbitrary, and the effects of a longer treatment protocol remain to be examined.

CpG emerged as an impressive antitumor TLR agonist in these studies, as previously described. Vicari et al. combined intratumoral CpG injections with systemic anti-IL10R antibody leading to the eradication of established B16F10 melanoma [4], an effect that depended upon endogenous CD40L [73]. Also, the combined administration of CpG and poly(I:C) synergistically elicited strong IL-12 production and antitumor activity against lung metastases [74]. However, there is a species difference for the expression of TLR9, the receptor for CpG, which is present on both myeloid and plasmacytoid DCs in the mouse but is restricted to plasmacytoid DCs in humans [75]. As a result of this difference, it has been argued that conclusions drawn from murine studies of CpG might be difficult to translate to human clinical trials. It is reassuring, therefore, that at least two pathways in humans have been shown to lead from CpG-initiated immunostimulation of TLR9+ plasmacytoid DCs to the activation of TLR9- myeloid DCs. Gerosa et al. found that CpG-activated human plasmacytoid DCs activated NK cells which in turn matured myeloid DCs for antigen presentation and for IL-12p70 production through a pathway that was at least partially dependent on NK cell-myeloid DC cell-cell contact [76]. Similarly, Gautier et al. found that type I interferon production from CpG-stimulated human plasmacytoid DCs could enhance IL-12p70 production by myeloid DCs [77].

The activity of poly(I:C) as an antitumor agent replicates many previous studies performed over the past 40 years [78]. While poly(I:C) is usually considered to be a dsRNA stimulator of TLR3 that leads to Type I interferon production, it is also an activator of MDA5, a helicase containing a CARD motif that can lead to IL-1beta processing and secretion [79]. The exact pathway by which poly(I:C) is acting in the present system remains to be established.

The histological studies that were performed are consistent with current concepts on how an intratumoral treatment might lead to tumor rejection. The apparent decrease in intratumoral CD11c+ DCs following treatment (Fig. 4B) is consistent with DC activation, a shift to CCR7 expression, and chemotaxis through lymphatics to the TDLNs [58]. Giuducci et al demonstrated such DC movements using FITC-labeled beads as a tracer within 6 hours of initiating immunotherapy with intratumoral AdCCL16 and systemic CpG and anti-IL-10R antibody in TSA breast tumors in mice [73]. The appearance of CD8+ T cells in the tumors is consistent with the recognized antitumor effects of these cytotoxic cells (Fig. 4C).

Finally, nanoparticles formed from plasmid DNA and cationic polymers such as PEI or C32 can play a crucial role in augmenting the effectiveness of certain immunostimulatory combinations. While both polymers augment plasmid-directed gene expression after intratumoral injection, C32 was shown to be significantly stronger than PEI, and also C32 lacks the cytotoxicity caused by PEI [34]. Using either PEI or C32, pSP-D-CD40L-containing nanoparticles were superior to control pcDNA3.1-containing nanoparticles when used in combination with CpG + poly(I:C) (Figs. 5 and 6).

However, there are fine differences between PEI and C32. PEI enhances the antitumor activity of pSP-D-CD40L by itself (compare pSP-D-CD40L naked DNA with PEI pSP-D-CD40L nanoparticles in Fig. 5). In contrast, C32 does not increase the antitumor activity of pSP-D-CD40L by itself (compare pSP-D-CD40L naked DNA with C32 pSP-D-CD40L nanoparticles in Fig. 6). There are two possible explanations for this difference. First, neither polymer is immunologically neutral. PEI was recently reported to be a strong TLR5 agonist that activates cells through a MyD88-dependent pathway [80], much like flagellin which is the prototypic TLR5 agonist. Likewise, C32 was shown to be an adjuvant for CD8+ T cell responses following intramuscular DNA vaccination using complexes of C32 and a plasmid for HIV gp120. In that case, C32 was used at a 2∶1 ratio of polymer to DNA rather than the 20∶1 ratio used in the present study [81]. Second, both cationic polymers are designed to help the plasmid DNA escape degradation in the lysosome and thereby favor the release of DNA into the cytoplasm. Such lysosomal damage could lead to activation of the NALP3 inflammasome pathway and result in the production of immunostimulants including IL-1β and IL-6 by dendritic cells [82]. In this regard, it is possible that PEI and C32 nanoparticles differ in their ability to activate the inflammasome pathway following intratumoral injection. As a further complexity, CD40L has been shown to downregulate NALP3-mediated inflammasome activation in macrophages, thereby suppressing the inflammasome-triggered release of inflammatory cytokines [83]. Perhaps this CD40-mediated inflammasome suppression is offset by the strong stimulation provided by CpG + poly(I:C) in a way that maintains the release of IL-1β and IL-6. Such complex interactions could underlie the need for combinations of immunostimulants to produce the strong antitumor effects observed in these experiments.

Antitumor immune responses are frequently associated with autoimmunity. In the case of B16F10 melanoma, autoimmunity takes the form of loss of pigment (vitiligo) caused by immune damage to normal melanocytes [31]. Although the combination therapy described here had significant antitumor effects on B16F10 melanoma, vitiligo was not seen. This is only the fifth report of a method to induce significant anti-melanoma immune responses without eliciting autoimmune vitiligo [84], [85], [86], [87]. The only toxic effect noted was a transient 2–3 fold increase in spleen size in the CpG treated mice consistent with previous reports that CpG induces extramedullary hematopoiesis in this organ [88].

In conclusion, the B16F10 melanoma model is an extremely difficult tumor to treat using immunotherapy approaches [89], which makes it an excellent system to identify immunotherapy formulations strong enough to translate into a clinical benefit for humans. Using this system, intratumoral injections of plasmid DNA encoding multimeric soluble CD40L (pSP-D-CD40L) demonstrated antitumor activity under certain circumstances and were especially effective when combined with TLR3 and TLR9 agonists. Many other TNF superfamily molecules have been found to have antitumor potential [90] and these could also be tested against B16F10 tumors using an adaptation of these methods (as an example, note the antitumor effects shown for pSP-D-GITRL in Fig. 1). The combination immunotherapy protocol described herein was non-toxic and did not elicit autoimmune effects such as vitiligo. A transient increase in spleen size related to CpG was the only negative effect detected. These results show the potential of immunostimulatory combinations for antitumor therapy and encourage further experiments to delineate the optimal use and role of each component in pSP-D-CD40L nanoparticle plus CpG and poly(I:C) approach.
